# Efficacy of 5-Nitroimidazoles for the Treatment of Giardiasis: A Systematic Review of Randomized Controlled Trials

**DOI:** 10.1371/journal.pntd.0002733

**Published:** 2014-03-13

**Authors:** Vinay Pasupuleti, Angel Arturo Escobedo, Abhishek Deshpande, Priyaleela Thota, Yuani Roman, Adrian V. Hernandez

**Affiliations:** 1 Department of Medicine, Case Western Reserve University, Cleveland, Ohio, United States of America; 2 Hospital Pediátrico Pedro Borrás Astorga, La Habana, Cuba; 3 Department of Medicine, Medicine Institute, Cleveland Clinic, Cleveland, Ohio, United States of America; 4 Unidad de Análisis y Generación de Evidencias en Salud Pública (UNAGESP), Instituto Nacional de Salud, Lima, Peru; 5 Postgraduate School, Universidad Peruana de Ciencias Aplicadas (UPC), Lima, Peru; 6 Health Outcomes and Clinical Epidemiology Section, Department of Quantitative Health Sciences, Lerner Research Institute, Cleveland Clinic, Cleveland, Ohio, United States of America; Universidad Nacional Autónoma de México, Mexico

## Abstract

**Background:**

Giardiasis is one of the most common causes of diarrheal disease worldwide and 5-nitroimidazoles (5-NI) are the most commonly prescribed drugs for the treatment of giardiasis. We evaluated the efficacy of 5-nitroimidazoles (5-NI) in the treatment of giardiasis in a systematic review of randomized controlled trials (RCTs).

**Methodology/Principal Findings:**

We conducted a comprehensive literature search in PubMed-Medline, Scopus, Web of Science and Cochrane Library for RCTs evaluating the efficacy of 5-NI vs. control (placebo or active treatment) on parasitological cure in patients with parasitologically-demonstrated giardiasis. The search was performed in May 2013 with no language restriction by two authors independently. The efficacy outcome was parasitological cure, and harmful outcomes were abdominal pain, bitter or metallic taste, and headache. We included 30 RCTs (n = 3,930). There was a significant and slightly higher response rate with 5-NI in giardiasis treatment (RR 1.06, 95%CI 1.02–1.11, p = 0.005). There was high heterogeneity among studies (I^2^ = 72%). The response rates for metronidazole, tinidazole and secnidazole were similar (RR 1.05, 95%CI 1.01–1.09, p = 0.01; RR 1.32 95%CI 1.10–1.59, p = 0.003; and RR 1.18 95%CI 0.93–1.449, p = 0.18, respectively). On subgroup analyses, the response rates did not vary substantially and high heterogeneity persisted (I^2^ = 57%–80%). Harmful outcomes were uncommon, and 5-NIs were associated with lower risk of abdominal pain, and higher risk of both bitter or metallic taste and headache.

**Conclusions:**

Studies investigating the efficacy of 5-NI in giardiasis treatment are highly heterogeneous. 5-NIs have a slightly better efficacy and worse profile for mild harmful outcomes in the treatment of giardiasis in comparison to controls. Larger high quality RCTs are needed to further assess efficacy and safety profiles of 5-NI.

## Author Summary

Giardiasis is a major diarrheal disease with worldwide distribution. 5-nitroimidazoles, which include metronidazole and tinidazole, are the most commonly used drugs in the treatment of giardiasis. In recent years, many other drugs with variable efficacies and adverse effects have been proposed for the treatment of giardiasis. No systematic review has evaluated efficacy of 5-nitroimidazoles as a group in comparison to the other antigiardial drugs. In this context, we performed a systematic review of the literature to identify randomized controlled trials comparing the efficacies of 5-nitroimidazoles with a control drug with the aim of assessing effectiveness of 5-nitroimidazoles in the treatment of giardiasis. Four research databases were searched; 30 trials with 3,930 subjects met our inclusion criteria. Results show that there was a high variation of study outcomes between included studies. 5-nitroimidazoles were associated with higher giardiasis cure rates than controls; also, 5-nitroimidazoles are associated with lower risk of abdominal pain, and higher risks of bitter or metallic taste and headache than controls.

## Introduction

Giardiasis is an intestinal illness caused by a flagellated protozoan parasite, *Giardia lamblia* (syn. *G.intestinalis and G.duodenalis*). The World Health Organization (WHO) estimates that 3 billion people reside in places with giardiasis prevalence of around 30%, and suggests that there are almost one billion cases of giardiasis, contributing to 2.5 million deaths annually from diarrheal disease [1]. In recent years, epidemiology of giardiasis in developed countries has been changing with increasing international travel and migration from highly endemic countries [2]. Approximately, 20,000 new cases of giardiasis are reported annually in the United States [3]. Due to its increasing global burden, and its developmental and socio-economic impact on infected individuals, *Giardia* has been included in the ‘Neglected Disease Initiative’ of the WHO since 2004 [4].

The most common antibiotics used for the treatment of giardiasis are the 5-Nitroimidazoles (5-NIs); these include metronidazole, tinidazole, secnidazole and ornidazole, of which metronidazole is the most common [5]. Alternative agents which are less commonly used in giardiasis treatment are quinacrine, furazolidone, benzimidazoles (albendazole and mebendazole), paromomycin, bacitracin zinc, chloroquine and nitazoxanide [5]. Depending on local epidemiology, availability, and cost, these drugs have been widely available for the curative treatment of cases; however, several reports of treatment failure have been reported [6], [7], [8]. With the advent of newer agents which might have similar efficacies as 5-NIs,and also offer an added advantage of more simplified regimens, fewer adverse effects or less drug resistance, it is of considerable interest to determine whether 5-NIs are still the best available option in the treatment of giardiasis.

Three previous systematic reviews and/or meta-analyses [9], [10], [11] have evaluated efficacies of antigiardial drugs in the treatment of giardiasis. All three varied in study designs and study aims, but none compared efficacy of 5-NIs as a group in comparison to other antigiardial drugs. Against this background, we performed a systematic review of the literature to identify RCTs comparing the efficacies of 5-NIs with a control with the aim of assessing effectiveness of 5-NIs in the treatment of giardiasis. We hope to provide policymakers and practitioners with a convenient and evidence-based summary of the primary literature on which to base their decisions.

## Methods

### Data sources and searches

A comprehensive literature search using PubMed-Medline from database inception through May 13, 2013, The Cochrane library from database inception through May 13, 2013, The Web of Science from database inception through May 13, 2013 and Scopus from database inception through May 13, 2013 was conducted by three investigators (AVH, VP and AD). The following keywords were used: metronidazole, tinidazole, secnidazole, ornidazole, 4-nitroimidazole, 5-nitroimidazole, *Giardia*, *G. lamblia*, giardiasis, randomized controlled trial and clinical trial.

### PubMed search strategy

((“metronidazole”[MeSH Terms] OR “metronidazole”[All Fields]) OR (“tinidazole”[MeSH Terms] OR “tinidazole”[All Fields]) OR (“secnidazole”[Supplementary Concept] OR “secnidazole”[All Fields]) OR (“ornidazole”[MeSH Terms] OR “ornidazole”[All Fields]) OR (“4-nitroimidazole”[Supplementary Concept] OR “4-nitroimidazole”[All Fields] OR “5 nitroimidazole”[All Fields])) AND ((“*Giardia*”[MeSH Terms] OR “*Giardia*”[All Fields]) OR *G.lamblia*[All Fields] OR (“giardiasis”[MeSH Terms] OR “giardiasis”[All Fields])) AND ((“randomized controlled trial”[Publication Type] OR “randomized controlled trials as topic”[MeSH Terms] OR “randomised controlled trial”[All Fields] OR “randomized controlled trial”[All Fields]) OR (“randomized controlled trial”[Publication Type] OR “randomized controlled trials as topic”[MeSH Terms] OR “randomized controlled trial”[All Fields] OR “randomised controlled trial”[All Fields]) OR (“clinical trial”[Publication Type] OR “clinical trials as topic”[MeSH Terms] OR “clinical trial”[All Fields]))

The following predetermined inclusion criteria were used: (i) RCTs evaluating the efficacy of 5-NI in comparison with a control (placebo, active treatment); (ii) study population of patients with parasitologically-demonstrated giardiasis; (iii) study in any language. An active treatment group is a control group receiving comparator drug (5-NI or non-5-NI) for the treatment of giardiasis. Our exclusion criteria were: (i) no control group; (ii) efficacy data (parasitological cure rates) were not available or could not extracted for the study groups.

### Study selection and data extraction

A list of retrieved articles was reviewed independently by 3 investigators (AVH, VP and AD) in order to choose potentially relevant articles, and disagreements about particular studies were discussed and resolved by consensus.

Two reviewers (VP and AD) independently extracted data from studies. The following information was extracted: age, gender, geographic location, study setting, diagnostic test for giardiasis, type of 5-NI and dose/duration, comparator drug and dose/duration, follow up time. Extracted beneficial outcome was parasitological cure rate and harmful outcomes were abdominal pain, bitter or metallic taste, and headache. One other author (AVH) reviewed the extractions for inconsistencies, and the three investigators (AVH, VP and AD) reached consensus.

### Evaluation of study quality

The quality of all included trials was assessed using a 5-item instrument developed and validated by Jadad [12]. The 5 items in this scale include i) description of randomization, ii) appropriateness of randomization, iii) description of blinding, iv) adequacy and appropriateness of blinding, and v) description of withdrawals and dropouts. Study quality was assessed independently by two investigators (VP and AVH). Disagreements were resolved by consensus. A score of 0–2 was considered as low quality trial and a score of 3–5 was considered high quality trial.

### Data synthesis and analysis

Our systematic review followed the Preferred Reporting Items for Systematic Reviews and Meta-Analyses (PRISMA) statement (Text S1, available as supporting data) [13]. A high degree of heterogeneity among studies was expected and therefore a formal meta-analysis was a secondary aim. Taking into account the sources of heterogeneity, several subgroup meta-analyses were pre-specified: (i) type of 5-NI used, (ii) excluding studies with two types of 5-NI comparisons, (iii) study setting (outpatient vs hospitalized), (iv) Jadad score (≥3 vs <3), (v) type of main analysis (intention-to-treat vs per-protocol), (vi) sample size (<100 vs ≥100 patients), (vii) ordered by year of publication. DerSimonian and Laird random effects models were used for meta-analyses [14]. We used the inverse variance (IV) or Mantel-Haenzel (MH) method to calculate pooled RRs and 95% CIs, depending on the absence or presence of scarce outcomes, respectively. When efficacy of two 5-NIs was compared, the 5-NI arm was the arm with the larger sample size. Statistical heterogeneity was evaluated with the Cochran χ^2^ and the I^2^ statistics. I^2^ values of 30–60% represented a moderate level of heterogeneity. A P value of <0.1 for χ^2^ was defined as indicating the presence of heterogeneity. To examine bias in the results of the meta-analyses, the Egger's test was used to evaluate asymmetry of the funnel plots. Asymmetry of the funnel plots should not be equated with publication bias, as asymmetry can be caused by true heterogeneity among study results, poor methodological quality, reporting biases, and chance. We used Review Manager (RevMan 5.0, Oxford, UK; The Cochrane Collaboration, 2008).

## Results

### Eligible studies

**Figure 1:**
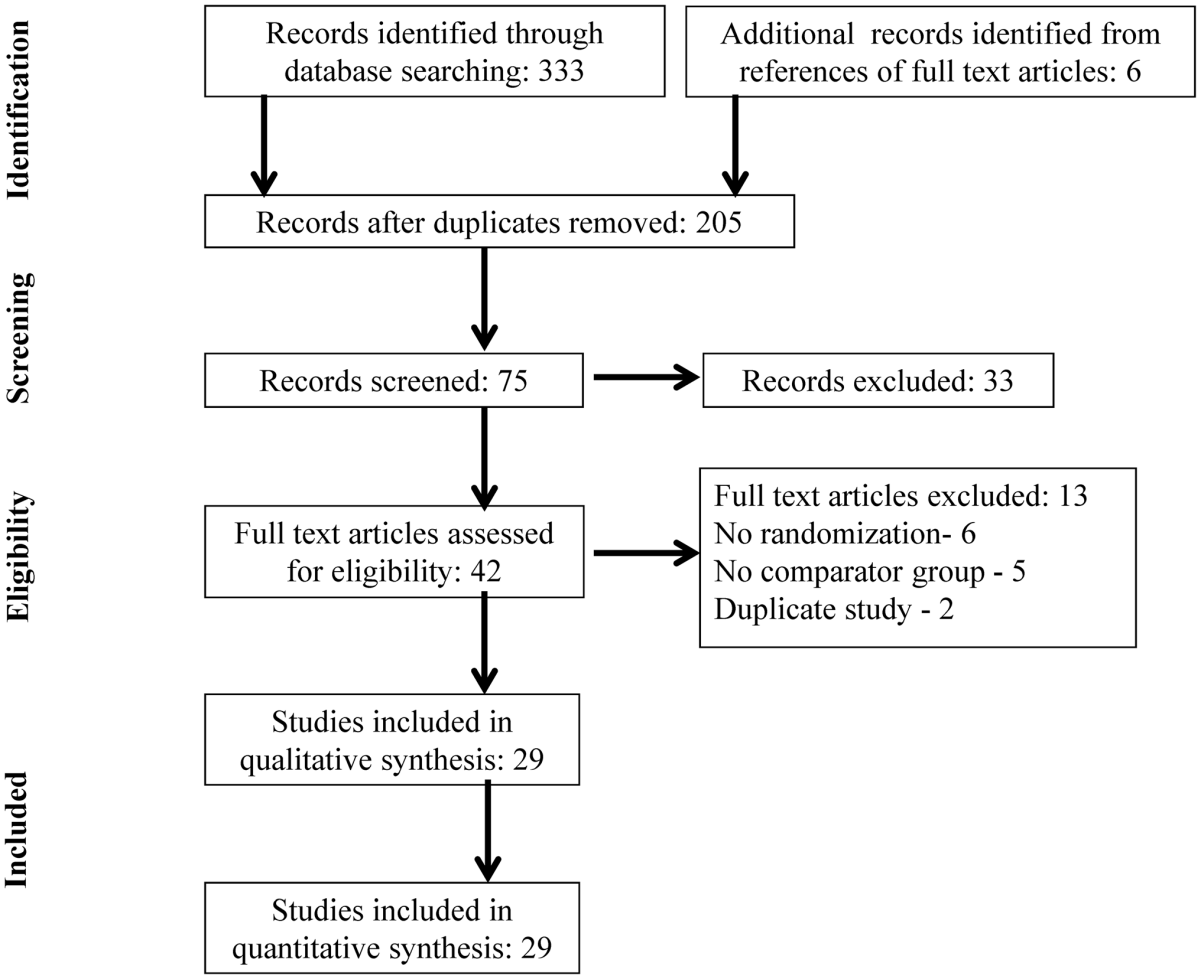
Flow diagram of selected studies.

Our search identified 333 publications (Figure 1). After removing duplicates and screening titles of the studies, 75 articles were selected based on relevance to the study topic. After screening the abstracts of these potentially relevant articles, 42 were selected for full-text review based on relevance to the study topic (Figure 1). Thirty randomized controlled trials (n = 3,930) in twenty-nine studies that reported efficacy data of 5-NIs in comparison to a control were included in the systematic review and meta-analyses. The reasons for exclusion of the remaining 13 articles are listed in Figure 1.

### Study characteristics

Table 1 summarizes the main characteristics of the included studies. All trials were conducted in countries endemic to giardiasis. Of the 30 trials included, 22 were in outpatient population; 7 in hospitalized population; one trial did not report the study setting. All patients included in the trials had parasitologically-demonstrated giardiasis. Twenty-four trials were in pediatric population (<18 yrs); 4 trials in both adult and pediatric population; two trials in patients of age >17 yrs. The post-treatment follow-up time varied from 3 days to 5 weeks. A total of 3,930 patients were included in the meta-analysis with sample size ranging from 23 to 502; all but one of the trials included active treatment controls (Table 1).

**Table 1:** Basic characteristics of included studies.

First author, Year published	Study location	Sample size	Study Population	Diagnostic test	Patient age category	Age Mean (SD)	Male (%)	Type of 5-NI, dose	Comparator drug, dose	Follow up time
al Waili NS, 1992 [22]	Iraq	44	NA	Stool exam for cysts and trophozoites	<18 yrs	NA	63.6	Metronidazole, 200 mg tid, 5 days	Mebendazole, 200 mg tid, 5 days	Day 7 & 14
Alizadeh A, 2006 [23]	Iran	120	outpatient	Trophozoites in iodine-stained wet stool preparations	Both	22.3 (11.0)	50.8	Metronidazole, 250 mg tid, 5 days	Albendazole, 400 mg daily, 5 days	10 days
Bassily S, 1970 [24]	Egypt	80	hospitalized	Stools by MIFC technique	Both	NA	NA	Metronidazole^€^, 250 mg bid daily, 10 days	Mepacrine^€^ 100 mg tid, 5 days; 2) Furazolidone^€^ 100 mg qd, 10 days; 3) Placebo 1 capsule tid, 7 days	5 wks
Bulut BU, 1996 [25]	Turkey	60	outpatient	Microscopic exam for parasites	<18 yrs	8.7 (1.6)	62.5	Metronidazole, 15 mg/kg daily, 7 days	Mebendazole, 100 mg tid, 7 days; 2) Mebendazole, 100 mg tid, 1 day, 3) Ornidazole, 40 mg/kg, single dose	2 wks
Canete R, 2006 [26]	Cuba	122	outpatient	Microscopic exam of fecal samples as wet mounts and/or after Ritchie concentration	<18 yrs	NA	51.6	Tinidazole, 50 mg/kg, single dose	Mebendazole, 200 mg tid, 1 day	Day 3, 5 & 7
Cimerman B, 1997 [27]	Brazil	267	outpatient	Proto-parasitologic feces test	<18 yrs	5.8 (2.8)	60.3	Tinidazole, 50 mg/kg, single dose	Secnidazole, 30 mg/kg, single dose	Day 7, 14 & 21
Dutta AK, 1994 [28]	India	150	hospitalized	Trophozoites/cyst in stool specimens	<18 yrs	NA	52.7	Metronidazole, 7.5 mg/kg/dose, tid 5 days	Albendazole, 400 mg once daily, 5 days	Day 1–7, 14 & 21
Escobedo AA, 2003 [29]	Cuba	146	outpatient	Microscopic exam of fecal samples as wet mounts and/or after Ritchie concentration	<18 yrs	8.3 (2.3)	54.8	Secnidazole, 30 mg/kg single dose	Mebendazole, 200 mg tid, 3 days	Day 3, 5 & 7
Escobedo AA, 2003 [30]	Cuba	165	outpatient	Stool exam as direct wet mounts and after formol-ether concentration	<18 yrs	6.4 (3.5)	52.7	Tinidazole, 50 mg/kg single dose	Chloroquine, 100 mg/kg bd, 5 days; 2) Albendazole, 400 mg daily, 5 days	Day 7 & 10
Escobedo AA, 2008 [31]	Cuba	166	outpatient	Microscopic exam of fecal samples as wet mounts and/or after Ritchie concentration	<18 yrs	7.8	52.4	Tinidazole, 50 mg/kg single dose	Nitazoxanide, 7.5 mg/kg bd, 3 days	Day 5 & 7
Fallah M, 2007 [32]	Iran	106	hospitalized	Stool exam by formalin ether concentration technique	<18 yrs	NA	65.1	Metronidazole, 15 mg/kg tid, 7 days	Tinidazole, 50 mg/kg single dose	1–2 wks
Gascon J, 1989 [33]	Spain	23	outpatient	Stool exam	>18 yrs	NA	73.9	Metronidazole, 250 mg tid, 7 days	Mebendazole, 200 mg tid, 1 day	Day 3, 7 & 30
Gazder AJ, 1978 [34]	India	100	hospitalized	Stool exam-hanging drop preparation as well as formalin-ether concentration technique	<18 yrs	5.5	65.0	Metronidazole, 50 mg/kg single dose	Tinidazole, 50 mg/kg single dose	Day 4, 8, 12 & 16
Hall A, 1993 [35]	Bangladesh	502	outpatient	Microscopic exam of stool-direct smear in saline and also fixed in 10% v/v formalin-saline and processed by ether sedimentation technique	<18 yrs	7.1	52.5	Metronidazole, 125 mg tid, 5 days	Albendazole, 400 mg once daily, 3 days; 2) Albendazole, 600 mg single dose	10 days
Hall A, 1993 [35]	Bangladesh	351	outpatient	Microscopic exam of stool-direct smear in saline and also fixed in 10% v/v formalin-saline and processed by ether sedimentation technique	<18 yrs	7.1	52.5	Metronidazole, 125 mg tid, 5 days	Albendazole, 400 mg once daily, 5 days; 2) Albendazole, 800 mg single dose	10 days
Karabay O, 2004 [36]	Turkey	67	outpatient	Stool exam for cysts/trophozoites	>18 yrs	39.5 (13.0)	42.1	Metronidazole, 500 mg tid, 5 days	Albendazole, 400 mg/day, 5 days	Day 7–15
Kavousi S, 1979 [37]	Iran	160	outpatient	Stool exam - direct smear and zinc sulfate concentration techniques	<18 yrs	5.4	53.8	Metronidazole, <2 yrs: 125 mg qd; 2–4 yrs: 125 mg bd, 5 days; 5–8 yrs: 125 mg tid, 5 days 9–13 yrs:250 mg tid, 5 days	Quinacrine, 8 mg/kg/day, 5 days	Day 5, 30 & 180
Leite EV, 1976 [38]	Brazil	30	hospitalized	Stool exam; centrifugation/fluctuation in zinc sulphate; centrifugation/sedimentation in formol-ether	Both	19 (1–63)*	66.6	Metronidazole, 1–3 yrs: 250 mg qd; 4–7 yrs: 250 mg bid; 8–12 yrs: 250 mg tid; Adults: 500 mg bid	Ornidazole, 1–3 yrs: 250 mg qd; 4–7 yrs: 250 mg bid; 8–12 yrs: 250 mg tid; Adults: 500 mg bid	10 days
Misra PK, 1995 [39]	India	64	hospitalized	Trophozoites and/or cysts in stool specimens	<18 yrs	NA	59.4	Metronidazole, 7.5 mg/kg/dose tid, 5 days	Albendazole, 400 mg once daily, 5 days	Day 1–7, 14 & 21
Ortiz JJ, 2001 [40]	Peru	110	outpatient	Cysts in stool	<18 yrs	5.7 (2.6)	49.1	Metronidazole, 125/250 mg bd, 5 days	Nitazoxanide, 100/200 mg bd, 3 days	7–10 days
Pengsaa K, 1999 [41]	Thailand	113	outpatient	Stool exam: direct smear in 0.9% saline and ether sedimentation method	<18 yrs	NA	52.9	Tinidazole, 50 mg/kg single dose	Albendazole, 400 mg once daily, 3 days	1–2 wks
Pine MC, 1999 [42]	Peru	79	outpatient	Parasitological examination	<18 yrs	7.8 (2.7)	NA	Metronidazole, 15 mg/kg/day tid, 10 days	1) Albendazole, 400 mg once daily, 5 days; 2) Furazolidone, 5 mg/kg/day qd, 10 days; 3) Tinidazole, 50 mg/kg/day single dose; 4) Secnidazole, 30 mg/kg/day, single dose	Day 7, 14 & 21
Quiros-Buelna E, 1989 [43]	Mexico	100	outpatient	Stool examination	<18 yrs	NA	NA	Metronidazole, <10 kg: 187.5 mg/day; 10–19.9 kg: 375 mg/day; 20–29.9 kg: 562.5 mg/day; ≥30 kg: 750 mg/day	Furazolidone, <10 kg: 66.6 mg/day; 10–19.9 kg: 133.2 mg/day; 20–29.9 kg: 199.8 mg/day ≥30 kg: 266.4 mg/day	Day 1–3
Romero-Cabello R, 1995 [44]	Mexico	100	outpatient	Stool exam, flotation/concentration method	<18 yrs	8 (4–11)^#^	49.0	Metronidazole, 7.5 mg/kg/dose tid, 5 days	Albendazole, 400 mg qd, 5 days	Day 1–7, 14 & 21
Sadjjadi SM, 2001 [45]	Iran	100	outpatient	Microscopic exam for ova/parasites by formalin-ether concentration technique	<18 yrs	NA	70.0	Metronidazole, 5 mg/kg/dose tid, 5 days	Mebendazole, 200 mg tid, 3 days	Day 7 & 14
Yereli K, 2004 [46]	Turkey	107	hospitalized	Saline-Lugol, formalin ethyl acetate concentration and trichrome staining	<18 yrs	8.3 (3.4)	47.7	Metronidazole, 6.7 mg/kg/dose tid, 7 days	Albendazole, 10 mg daily, 5 days	Day 7, 14 & 21
Canete R, 2010 [47]	Cuba	122	outpatient	Microscopic exam of fecal samples, as direct wet mounts and/or after Ritchie concentration	<18 yrs	NA	NA	Metronidazole, 5 mg/kg/dose tid, 5 days	Chloroquine, 10 mg/kg bd, 5 days	Day 3, 5 and 10
Teles NSB, 2011 [48]	Brazil	100	outpatient	Hoffman and Ritchie sedimentation method	Both	18.7 (13.4)	54.0	Secnidazole, Adults: 2000 mg single dose; Children: 30 mg/kg	Mentha crispa, 2000 mg single dose	7 days
Almirall P, 2011 [49]	Cuba	126	outpatient	Examination of fecal samples as direct wet mounts and/or after Ritchie concentration	>17 yrs	35.0 (13.5)	69.1	Secnidazole, 2000 mg single dose	Mebendazole, 200 mg tid, 3 days	Day 3, 5 & 10
Canete R, 2012 [50]	Cuba	150	outpatient	microscopic exam of fecal samples, as direct wet mounts and/or after Ritchie concentration	>18 yrs	29.5	48.7	Metronidazole, 250 mg tid, 5 days	Albendazole, 400 mg once daily, 5 days	Day 3, 5 & 7

NA  =  not available; *  =  Mean (range); ^#^  =  median (range); ^€^  =  children got half the adult dose.

### Study quality assessment and publication bias

**Figure 2:**
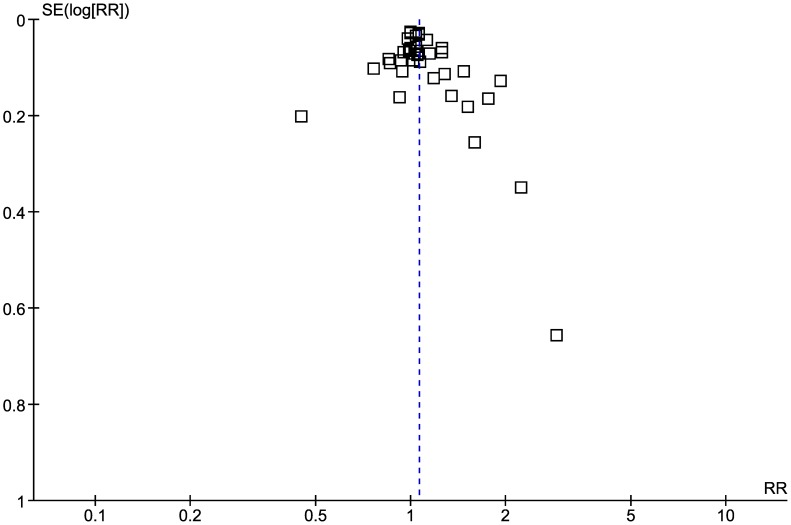
Funnel plot assessing publication bias.

Using the Jadad scale, 13 trials were identified as high quality (Table S1, available as supporting data). All studies were described as randomized, 13 of them appropriately described the generation of the sequence of randomization, but none of studies were appropriately blinded. Twenty-nine studies appropriately described withdrawal and dropouts. Publication bias assessed by funnel plot showed some asymmetry around the point estimate, especially for small sample size studies indicating presence of bias (Figure 2). The Egger test did not suggest asymmetry of the funnel plot (p = 0.1).

### Efficacy of 5-NIs in the treatment of giardiasis and meta-analyses of subgroups of studies

**Figure 3:**
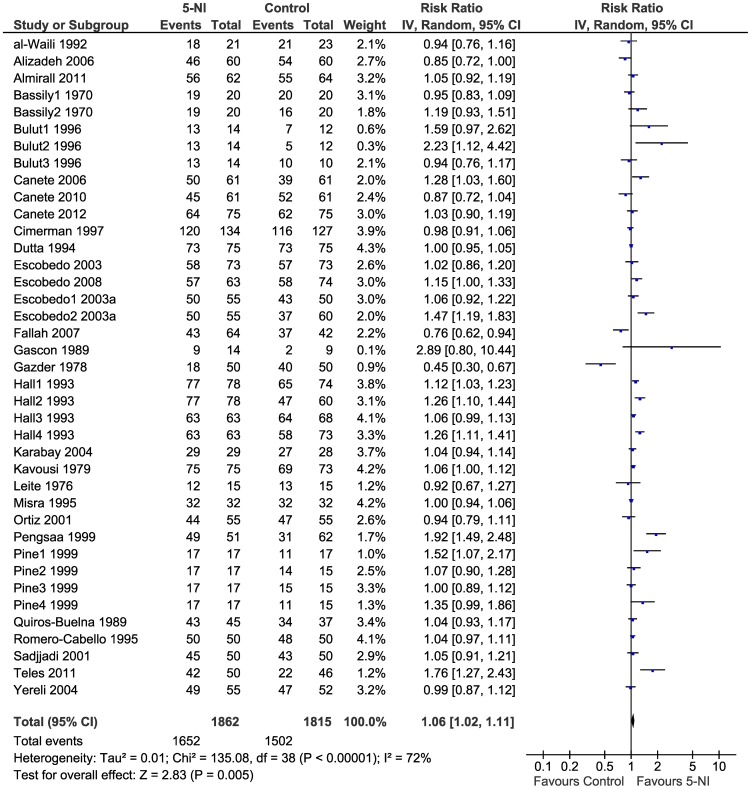
Forest plot showing efficacy of 5-NIs in the treatment of giardiasis.

**Figure 4:**
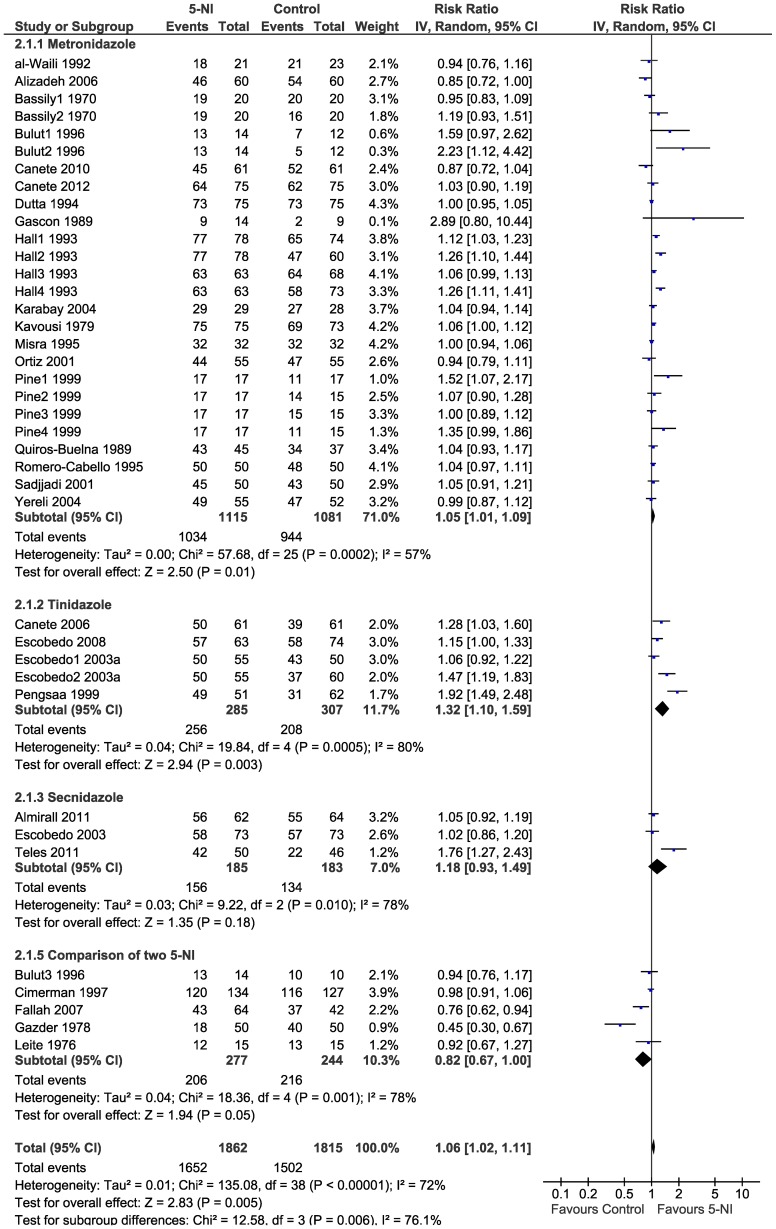
Forest plot showing efficacy of 5-NIs in the treatment of giardiasis; stratified by type of drug.

We found a significant and slightly higher cure rates (RR 1.06, 95%CI 1.02–1.11, p = 0.005) (Figure 3). There was high heterogeneity among studies (I^2^ = 72%). When stratified by type of drug, efficacy of 5-NIs did not vary significantly and high heterogeneity persisted (I^2^ = 57%–80%): metronidazole (RR 1.05, 95%CI 1.01–1.09, p = 0.01); tinidazole (RR 1.32 95%CI 1.10–1.59 p = 0.003); and secnidazole (RR 1.18 95%CI 0.93–1.49 p = 0.18) (Figure 4). There was no study comparing the drug ornidazole to a control group.

On excluding studies comparing two 5-NIs the pooled risk ratio did not vary significantly (RR 1.08, 95%CI 1.04–1.13, p<0.0001) (Figure S1). On subgroup analysis based on study setting, 5-NI efficacy in outpatients was RR 1.10 95%CI 1.05–1.15 p<0.0001 and in hospitalized patients was RR 0.94 95%CI 0.87–1.03 p = 0.18 (Figure S2). When only high quality studies (Jadad score ≥3) were pooled, 5-NI efficacy was RR 1.07 95%CI 1.02–1.13 p = 0.01 (Figure S3). When trials with ITT analysis were pooled RR was 1.01, 95%CI 0.96–1.06 p = 0.62 and trials with PP analysis were pooled RR was 1.15, 95%CI 1.07–1.23 p = 0.0001 (Figure S4). On pooling data from only large sample size studies (n >100), 5-NI efficacy was RR 1.06, 95%CI 1.01–1.12, p = 0.02 (Figure S5). When all trials were pooled in the order of year of publication no significant shift was observed in the trend of 5-NI efficacy in treatment of giardiasis over the years (Figure S6).

### Harmful outcomes associated with the use of 5-NI

Abdominal pain, bitter or metallic taste and headache were uncommon harmful outcomes. The use of 5-NI was associated with a lower risk of abdominal pain (RR 0.72, 95%CI 0.57–0.91; p = 0.007, I^2^ = 51%; Figure S7), higher risk of bitter or metallic taste (RR 3.27, 95%CI 2.66–4.01; p<0.00001, I^2^ = 100%; Figure S8), and higher risk of headache (RR 1.97, 95%CI 1.37–2.83; p = 0.0003, I^2^ = 46%; Figure S9).

## Discussion

We found that RCTs evaluating the efficacy of 5-NIs for giardiasis treatment are highly heterogeneous in terms of study design and outcomes. Heterogeneity could not be diminished after performing several pre-specified subgroup analyses. The quality of RCTs is mostly low, especially because of lack of double blinding. 5-NIs are associated with a slightly higher giardiasis cure rates than controls; also, 5-NIs are associated with lower risk of abdominal pain, and higher risks of bitter or metallic taste and headache than controls.

Since the first publication of metronidazole in the treatment of giardiasis by Schneider *et al* [15] more than 50 years ago, 5-NI compounds (mainly metronidazole and tinidazole) have become an important component of the antigiardial armamentarium in many parts of the world, owing to their efficacy, relative safety, universal availability, and cost-effectiveness. However, during these last five decades, 5-NI compounds, principally metronidazole, have also been usually prescribed for several other indications, including gingivitis, bacterial vaginitis, part of the combination treatment for *H. pylori*, infections with *Clostridium difficile* and other anaerobic bacteria, amebiasis, and as prophylaxis in colorectal surgery [16], [17].This wide spectrum usage of 5-NIs could have led to an increased occurrence of *G. lamblia* resistance; in fact, *Giardia* resistance towards common antigiardials has been demonstrated or induced *in vitro* [18] and also cross-resistance between metronidazole and tinidazole has been demonstrated [19]. Most of the therapeutically used antigiardial drugs, including metronidazole cause severe side effects and are not well tolerated by many patients and clinical resistance to medication has been observed for all common drugs in up to 20% of giardiasis cases. Treatment failure may be due to both host factors (e.g. low patient compliance due to side effects) and parasite resistance [5], [20]. The present study systematically reviewed all available data from trials examining parasitological outcomes, and comparing efficacy of different 5-NI drugs, doses and regimens. The results obtained, suggest that 5-NI continue to be efficacious for giardiasis. However, it should be stated that we did not find any trial comparing ornidazole to a control group, and so we cannot make recommendations for this drug.

Three previous systematic reviews on the treatment of giardiasis have been published (Table S2). In a systematic review by Zaat and colleagues [10], 31 RCTs published up to 1997 were included (n = 2,988). Three databases were searched, pseudo-randomized trials were included and no language restriction was used. Any trial of treatment of giardiasis comparing drugs or treatment regimens with placebo or other drugs/regimens were included. Metronidazole was found to be equally effective in parasitological cure as other longer therapies such as furazolidone. Tinidazole seems to be the most effective single-dose therapy in terms of parasitological cure compared to other short therapies, having, at the same time, relatively fewer harmful effects such as diarrhea at the end of follow-up. Trials were also found to be clinically and statistically heterogenous. Majority of the included studies were low on methodological quality as per Cochrane Collaboration guidelines. A recent meta-analysis by Solaymani-Mohammadi *et al* [11] included 8 RCTs (n = 900) published up to 2010 comparing effectiveness and safety of metronidazole vs. albendazole for the treatment of giardiasis. Six databases were searched, and no language restriction was applied. Effectiveness of albendazole was found to be comparable to metronidazole. Patients treated with albendazole also tended to have fewer side effects such as metallic taste and anorexia. Included trials were found to have moderate heterogeneity of effects and the quality was low in 7 of them (Jadad score 0–2).

A recent systematic review by Granados *et al* [9] included 19 studies (n = 1,817) published up to 2012 and evaluated the relative effectiveness of alternative antibiotic regimens for treating adults or children with symptomatic giardiasis. Six databases were searched, and no language restriction was used. All RCTs comparing metronidazole administered for five to 10 days with any of the following drugs: metronidazole (single dose), tinidazole, albendazole, mebendazole, and nitazoxanide were included. The primary outcomes were parasitological and clinical cure. They concluded that albendazole may be of similar effectiveness to metronidazole, may have fewer gastrointestinal and neurological side effects, and has the advantage of a simplified regimen; evidence was considered to be of moderate quality based on GRADE methodology. Included trials had moderate heterogeneity of effects.

Our study is the first to examine the efficacy of 5-NIs as a group in comparison to other antigiardial drugs in the treatment of giardiasis. Search criteria in our review were not restricted by language thereby avoiding ‘tower of babel bias’ [21]. Several RCTs included in our meta-analysis were deficient in quality (i.e. high risk of bias) in included trials. Given that most of studies did not appropriately use blinding, the probability of information bias and inflation of efficacy of 5-NIs is present. Also, since heterogeneity among studies was expected, we had pre-specified an extensive list of specific study variables for subgroup analyses (Figure S1–S5). On subgroup analysis both low quality studies and high quality studies (by Jadad scoring) showed higher efficacy of 5-NIs vs. controls, while only results from high quality studies achieved significance. Effect sizes remained fairly constant on the rest of subgroup analyses suggesting that heterogeneity of included trials might not have adversely affected our results. In our meta-analysis 28 trials included pediatric population and 6 trials included adult population. Including subjects of different age groups as well as with varied clinical manifestations and a variety of clinical settings allows us to generalize our findings of effectiveness of 5-NI treatment to all age groups and all kinds of symptomatic giardiasis. 5-NIs were associated with higher risk of bitter or metallic taste and headache. Though, these would be considered as minor adverse effects, drug tolerability and adverse events could potentially impact patient compliance. Our findings suggest that 5-NIs have high cure rates and reasonable safety profiles and in absence of better alternative drugs remain the drug the choice in the treatment of giardiasis.

Our study has specific limitations that need to be considered in the interpretation of our findings. There was heterogeneity in some of the relevant study design aspects (time of follow-up, different doses of the drugs, heterogeneity of participants, number of parasitological exams at follow-up, and laboratory techniques across the studies) which made some results difficult to interpret and precluded us from a more confident and robust conclusions about benefits and harms associated with the use of 5-NIs in the treatment of giardiasis. The Egger's test did not suggest asymmetry of the forest plot, but some degree of publication bias can be expected. Results should also be interpreted with caution in light of high proportion of low quality RCTs. Also, we used one of the several tools to evaluate risk of bias, the Jadad score. Unfortunately, this tool does not evaluate other potential and important sources of bias such as concealment of the randomized allocation and selective reporting of outcomes. Despite these limitations, this present review represents the most up-to-date, comprehensive, and systematic attempt to assess the efficacies of 5-NI drugs as in group in the treatment of giardiasis.

Studies investigating the efficacy of 5-NIs in the treatment of giardiasis are highly heterogenous. Though information available from RCTs on the use of 5-NI allow us to confirm that these drugs are still a good option for the treatment of giardiasis, there is a need for well designed, high quality RCTs to further assess safety and efficacy profiles of 5-NIs.

## Supporting Information

Figure S1 Forest plot showing efficacy of 5-NIs in the treatment of giardiasis; excluding studies comparing two 5-NI.
(TIFF)

Figure S2 Forest plot showing efficacy of 5-NIs in the treatment of giardiasis; stratified by type of patient (outpatient vs hospitalized).
(TIF)

Figure S3 Forest plot showing efficacy of 5-NIs in the treatment of giardiasis; stratified by Jadad score (≥3 vs <3).
(TIF)

Figure S4 Forest plot showing efficacy of 5-NIs in the treatment of giardiasis; stratified by type of main analysis (ITT vs PP).
(TIF)

Figure S5 Forest plot showing efficacy of 5-NIs in the treatment of giardiasis; stratified by sample size (<100 vs ≥100 patients).
(TIF)

Figure S6 Forest plot showing efficacy of 5-NIs in the treatment of giardiasis; ordered by year of publication.
(TIF)

Figure S7 Forest plot showing abdominal pain associated with 5-NI in the treatment of giardiasis.
(TIF)

Figure S8 Forest plot showing bitter or metallic taste associated with 5-NI in the treatment of giardiasis.
(TIF)

Figure S9 Forest plot showing headache associated with 5-NI in the treatment of giardiasis.
(TIF)

Table S1 Jadad scoring of included studies.
(DOC)

Table S2 Description of systematic reviews on drugs for treating giardiasis.
(DOCX)

Text S1 PRISMA guidelines checklist.
(DOC)
