# Supplementary figures and images for: Efficacy of 5-Nitroimidazoles for the Treatment of Giardiasis: A Systematic Review of Randomized Controlled Trials
Source: PLoS Negl Trop Dis. 2014 Mar 13;8(3):e2733. doi: 10.1371/journal.pntd.0002733 (PMC3953020; doi:10.1371/journal.pntd.0002733)

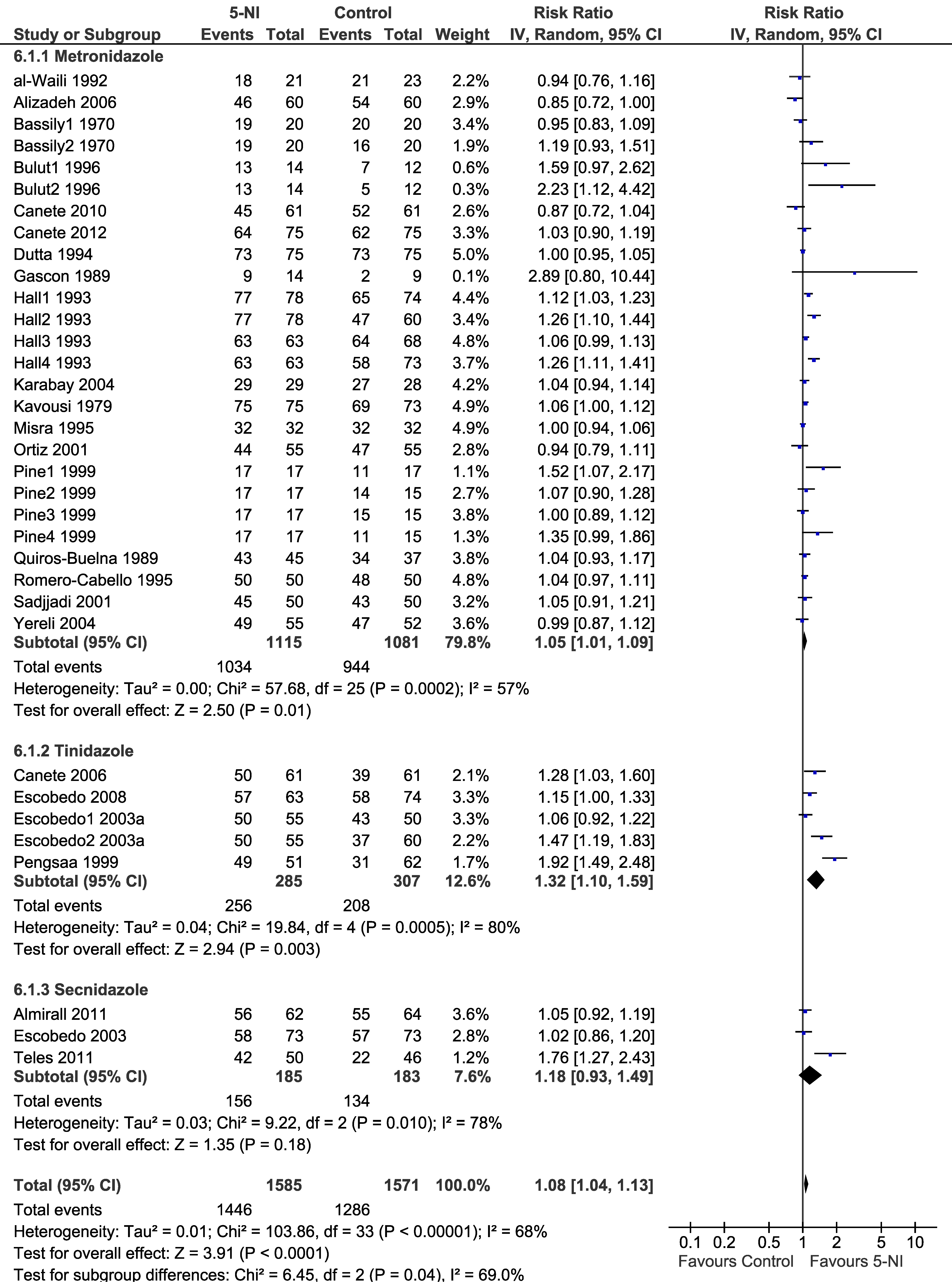

Supplement: Figure S1 — Forest plot showing efficacy of 5-NIs in the treatment of giardiasis; excluding studies comparing two 5-NI. (TIFF) [file pntd.0002733.s001.tif]

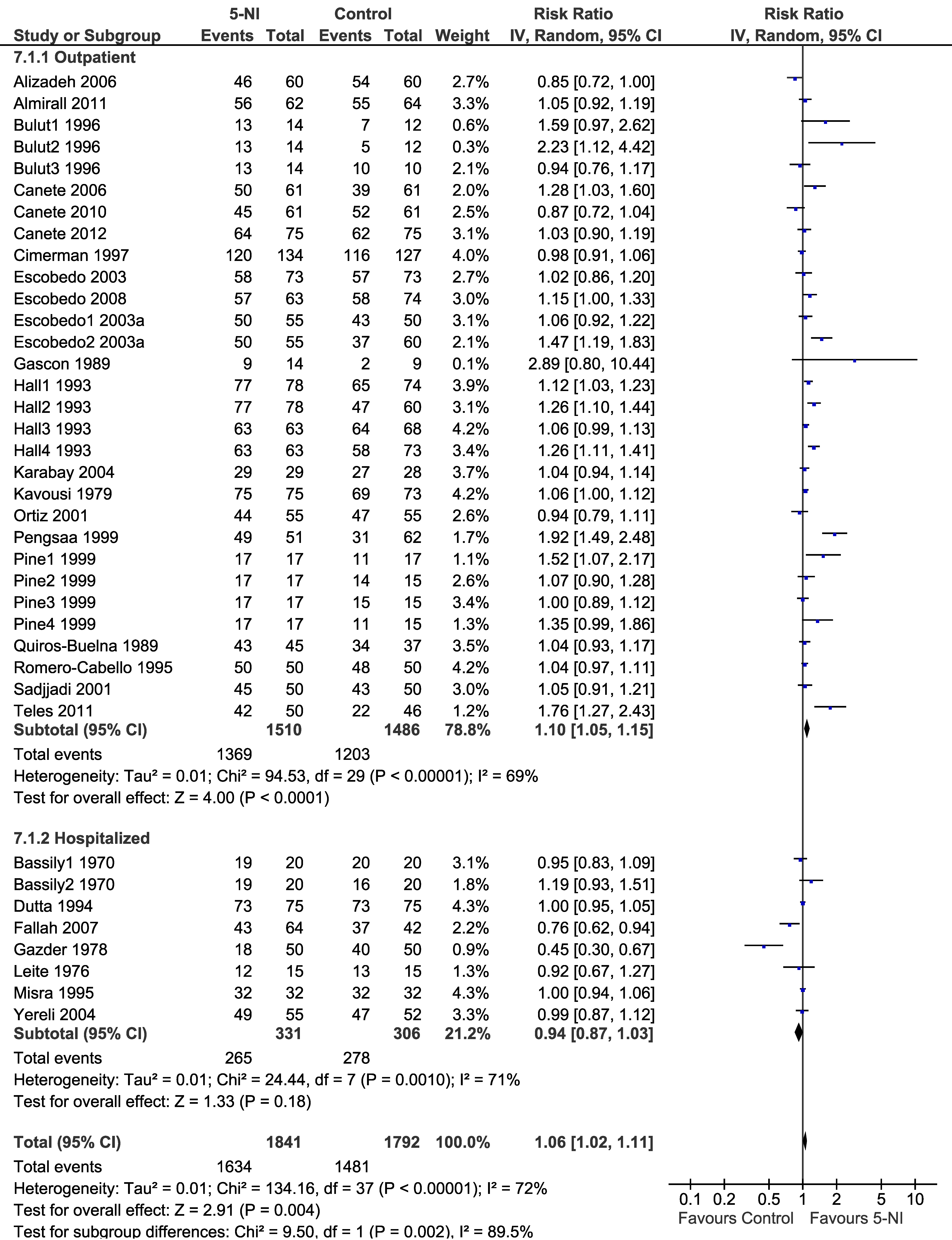

Supplement: Figure S2 — Forest plot showing efficacy of 5-NIs in the treatment of giardiasis; stratified by type of patient (outpatient vs hospitalized). (TIF) [file pntd.0002733.s002.tif]

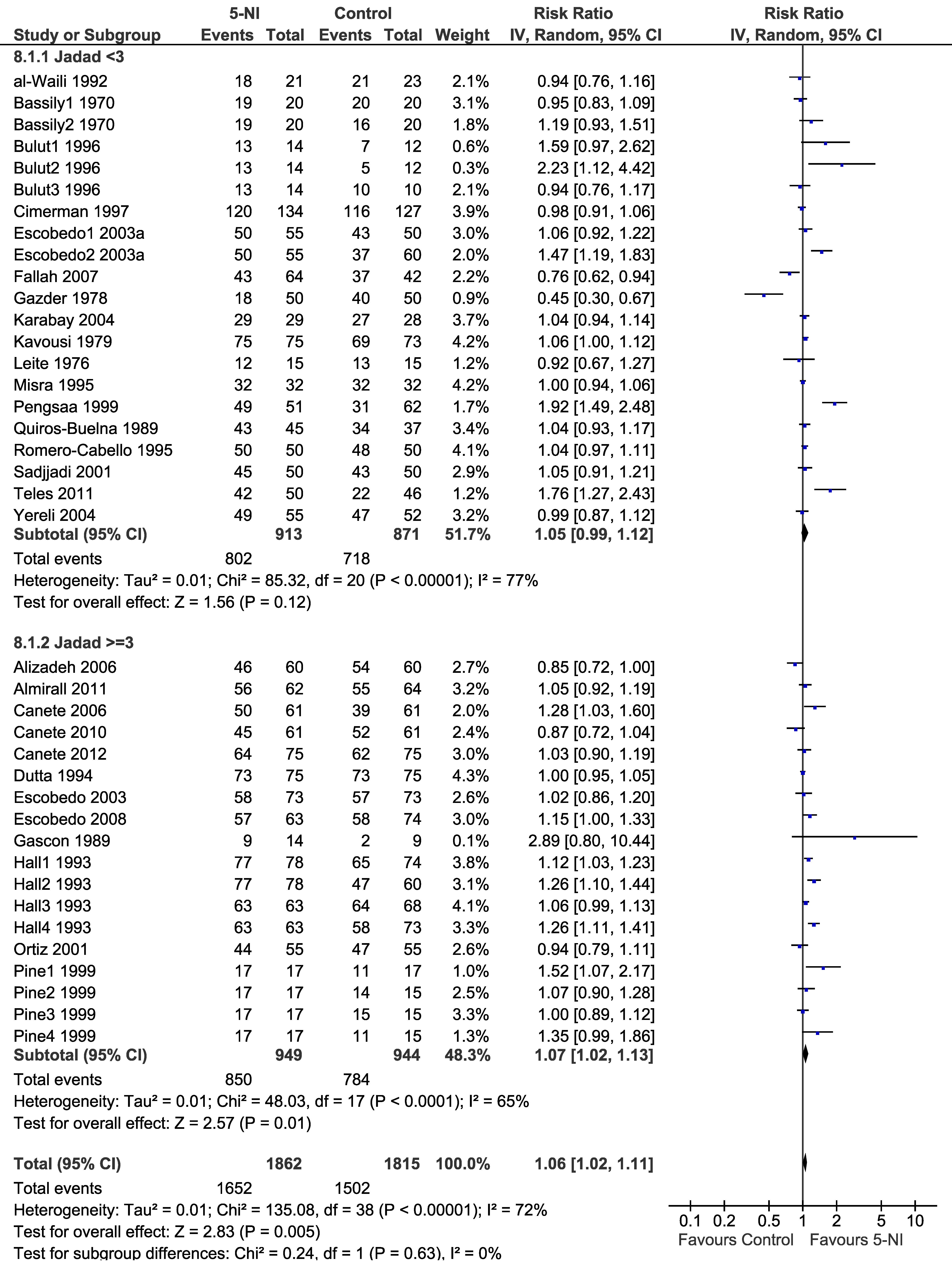

Supplement: Figure S3 — Forest plot showing efficacy of 5-NIs in the treatment of giardiasis; stratified by Jadad score (≥3 vs <3). (TIF) [file pntd.0002733.s003.tif]

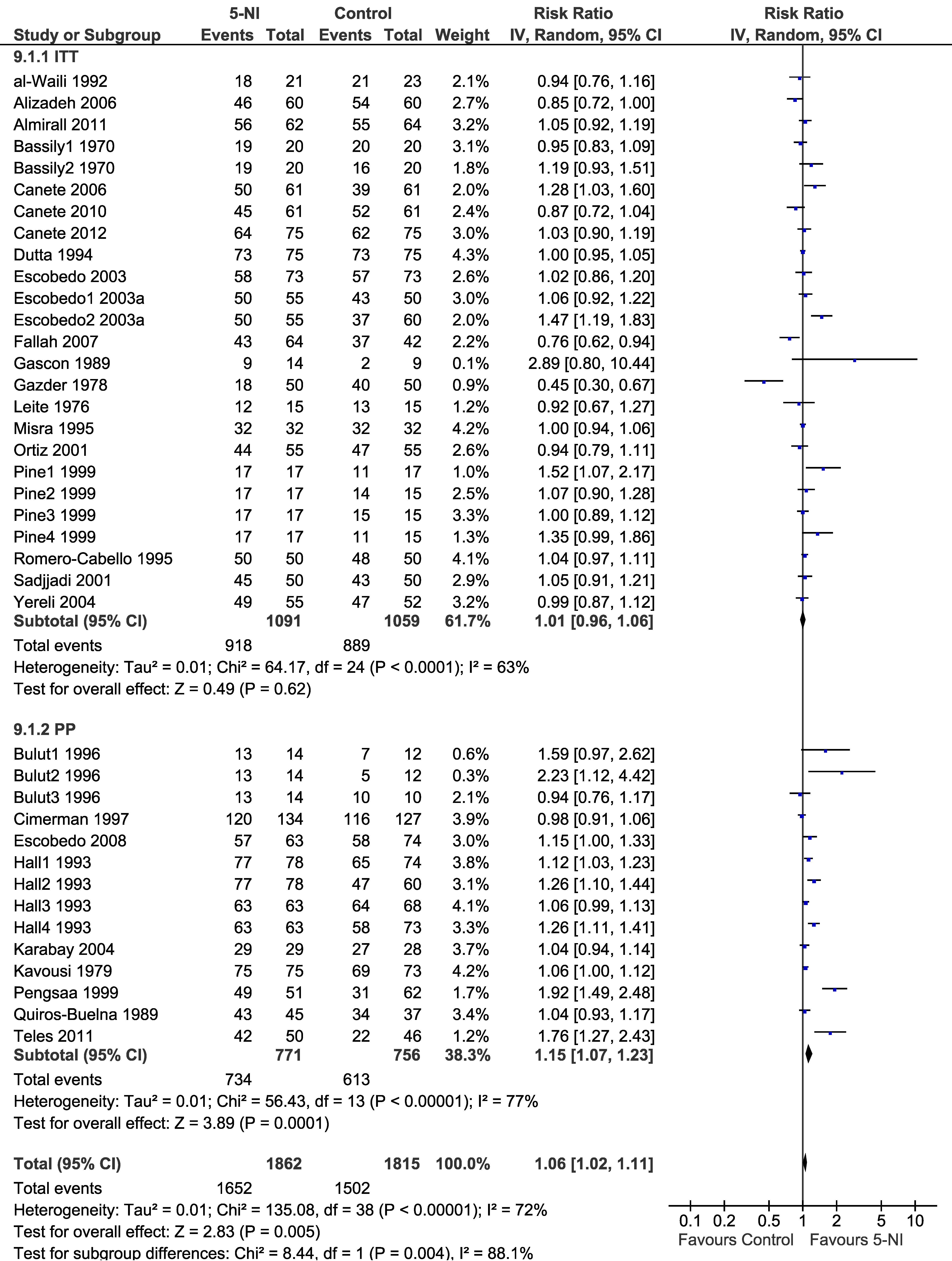

Supplement: Figure S4 — Forest plot showing efficacy of 5-NIs in the treatment of giardiasis; stratified by type of main analysis (ITT vs PP). (TIF) [file pntd.0002733.s004.tif]

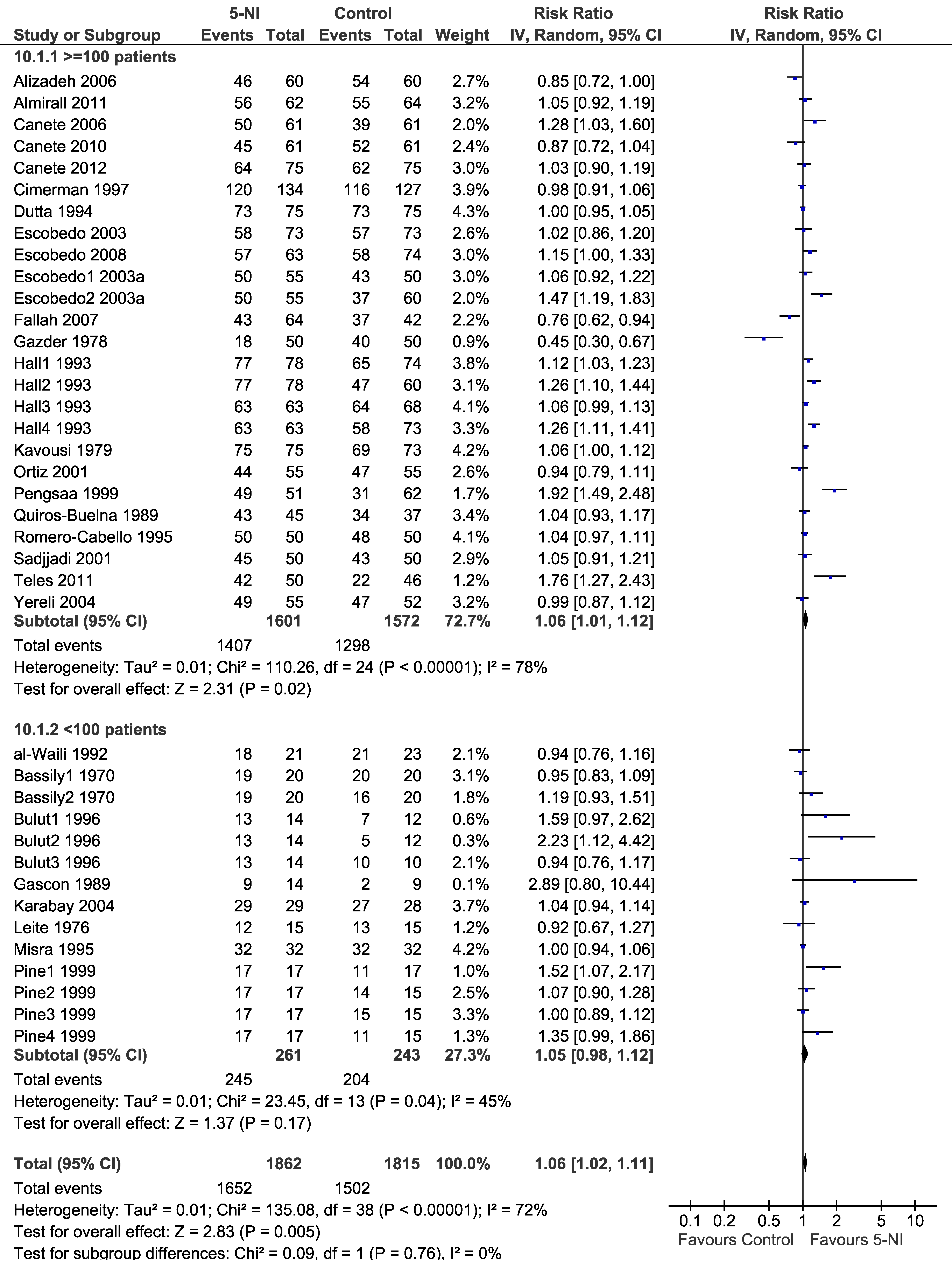

Supplement: Figure S5 — Forest plot showing efficacy of 5-NIs in the treatment of giardiasis; stratified by sample size (<100 vs ≥100 patients). (TIF) [file pntd.0002733.s005.tif]

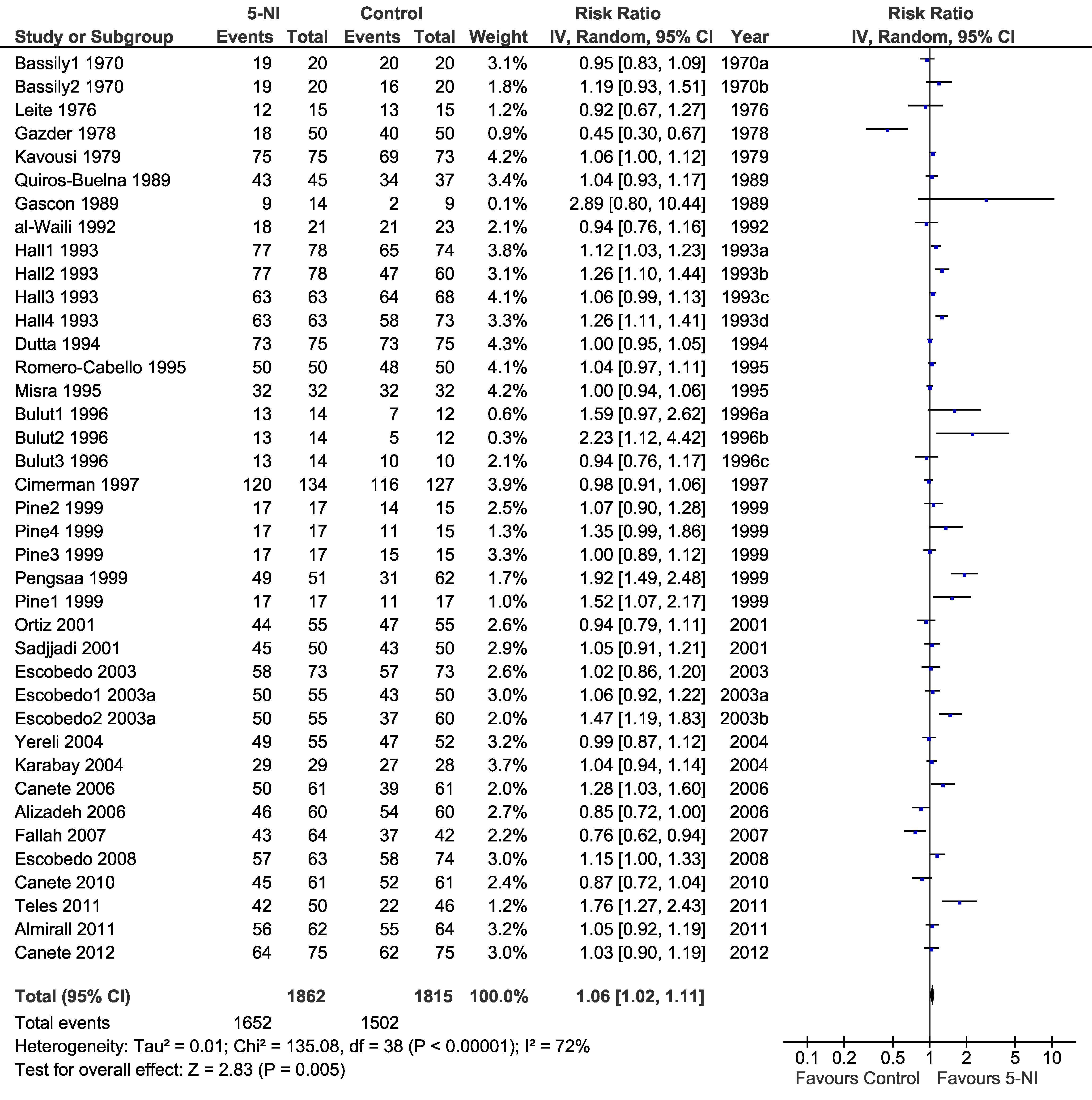

Supplement: Figure S6 — Forest plot showing efficacy of 5-NIs in the treatment of giardiasis; ordered by year of publication. (TIF) [file pntd.0002733.s006.tif]

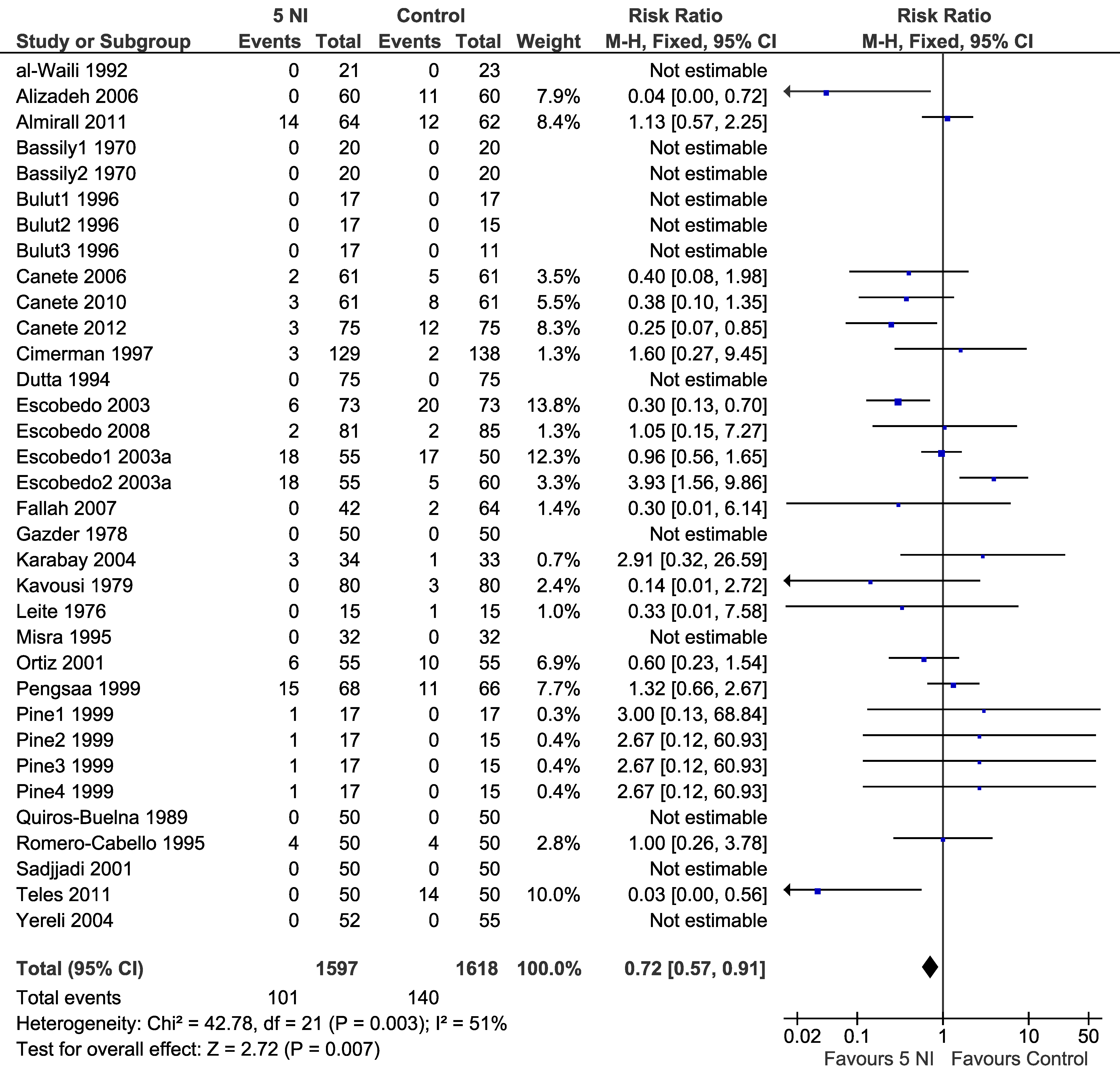

Supplement: Figure S7 — Forest plot showing abdominal pain associated with 5-NI in the treatment of giardiasis. (TIF) [file pntd.0002733.s007.tif]

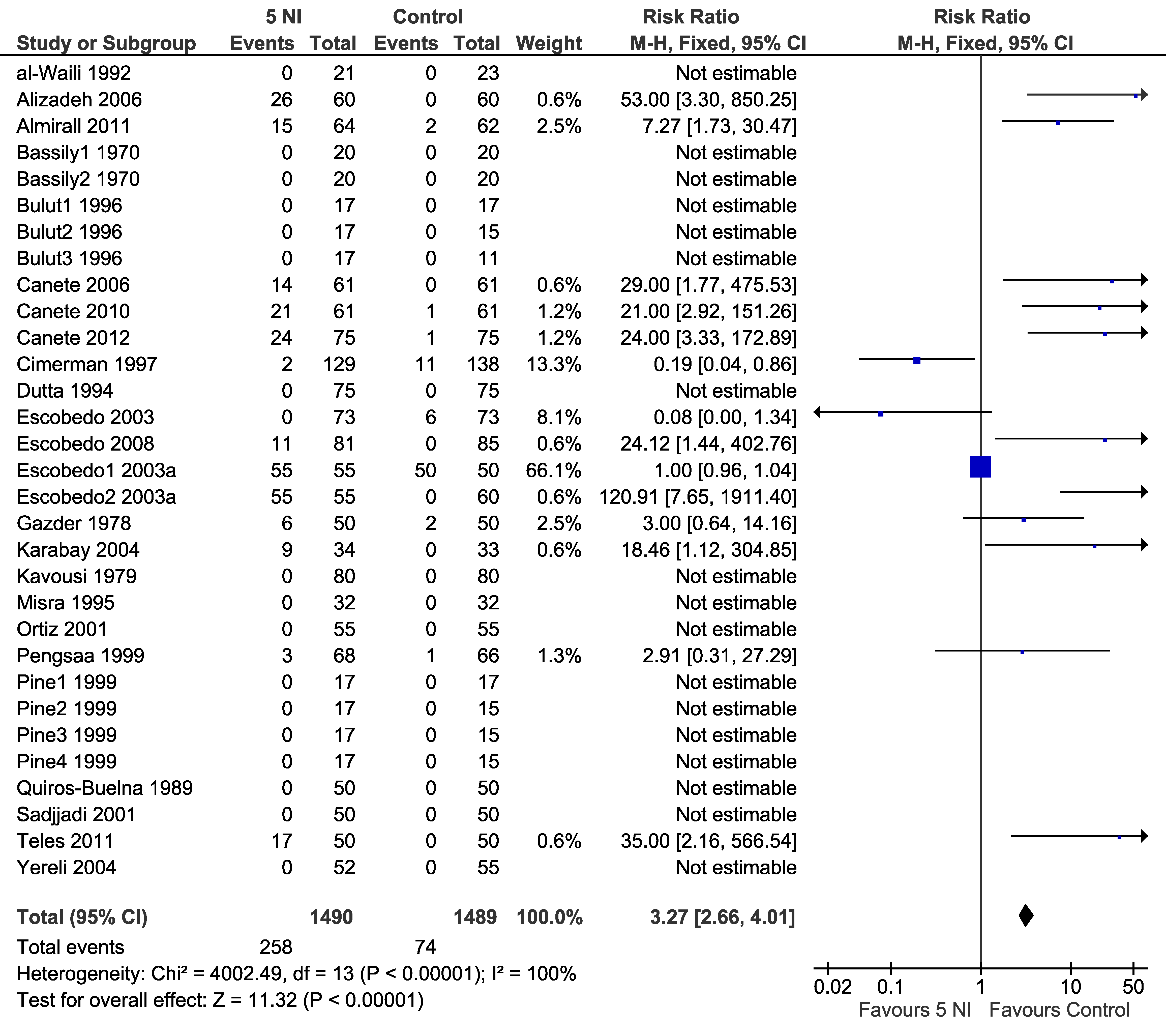

Supplement: Figure S8 — Forest plot showing bitter or metallic taste associated with 5-NI in the treatment of giardiasis. (TIF) [file pntd.0002733.s008.tif]

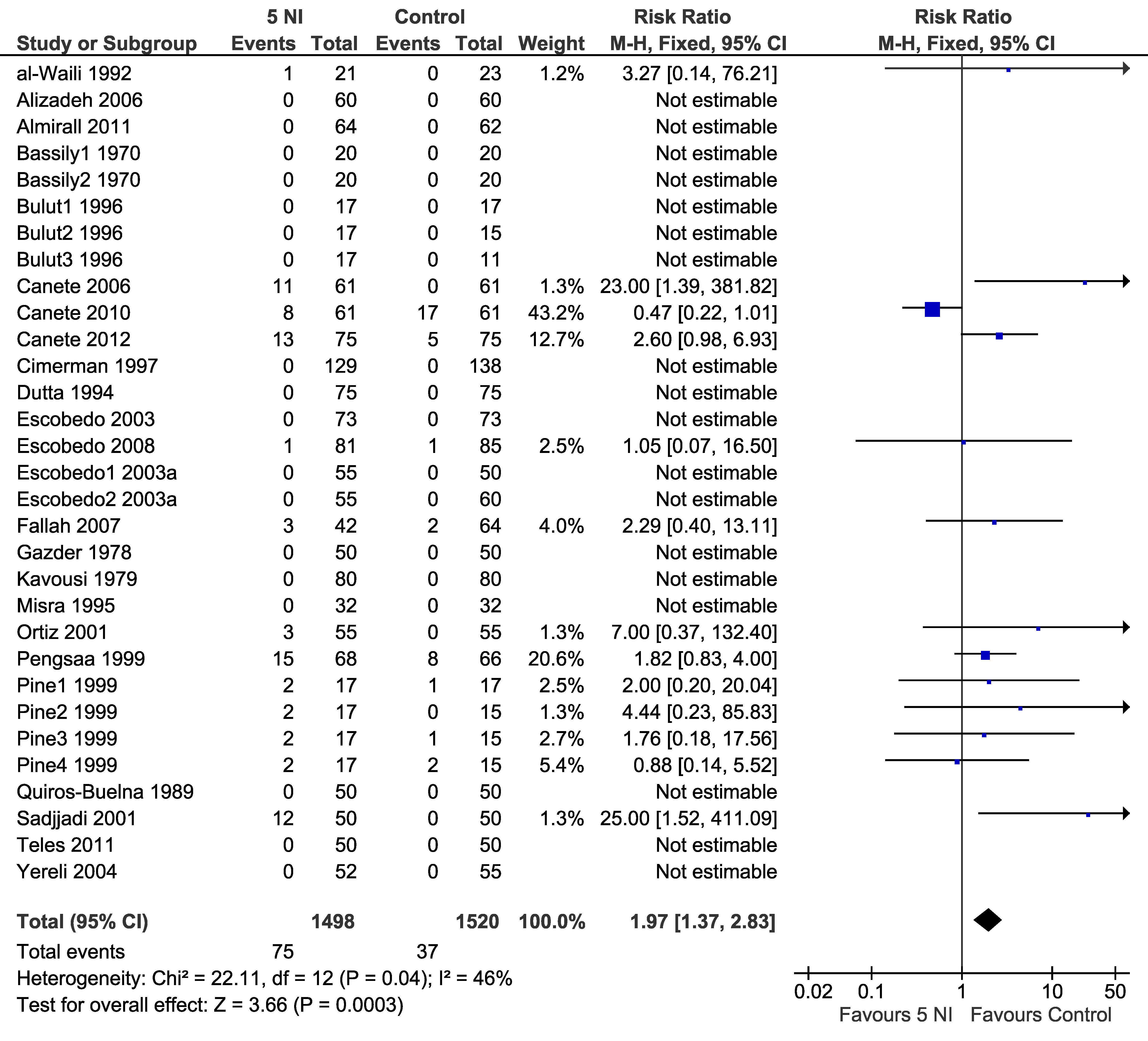

Supplement: Figure S9 — Forest plot showing headache associated with 5-NI in the treatment of giardiasis. (TIF) [file pntd.0002733.s009.tif]
