# Supplementary material for: Efficacy of 5-Nitroimidazoles for the Treatment of Giardiasis: A Systematic Review of Randomized Controlled Trials
Source: PLoS Negl Trop Dis. 2014 Mar 13;8(3):e2733. doi: 10.1371/journal.pntd.0002733 (PMC3953020; doi:10.1371/journal.pntd.0002733)
Supplement: Table S1 — Jadad scoring of included studies. (DOC) [file pntd.0002733.s010.doc]

| **Table S1**: Jadad scoring of included studies | | | | | | |
| --- | --- | --- | --- | --- | --- | --- |
| **First author** | **Study described as randomized** | **Method used to generate the sequence of randomization described and appropriate** | **Study described as double blind** | **Method of double blinding described and appropriate** | **Study described withdrawals and dropouts** | **Total Jadad score** |
| al-Waili NS | 1 | 0 | 0 | 0 | 1 | 2 |
| Alizadeh A | 1 | 1 | 0 | 0 | 1 | 3 |
| Bassily S | 1 | 0 | 0 | 0 | 1 | 2 |
| Bulut BU | 1 | 0 | 0 | 0 | 1 | 2 |
| Canete R | 1 | 1 | 0 | 0 | 1 | 3 |
| Cimerman B | 1 | 0 | 0 | 0 | 1 | 2 |
| Dutta AK | 1 | 1 | 0 | 0 | 1 | 3 |
| Escobedo AA | 1 | 1 | 0 | 0 | 1 | 3 |
| Escobedo AA | 1 | 0 | 0 | 0 | 1 | 2 |
| Escobedo AA | 1 | 1 | 0 | 0 | 1 | 3 |
| Fallah M | 1 | 0 | 0 | 0 | 1 | 2 |
| Gascon J | 1 | 1 | 0 | 0 | 1 | 3 |
| Gazder AJ | 1 | 0 | 0 | 0 | 1 | 2 |
| Hall A | 1 | 1 | 0 | 0 | 1 | 3 |
| Karabay O | 1 | 0 | 0 | 0 | 1 | 2 |
| Kavousi S | 1 | 0 | 0 | 0 | 1 | 2 |
| Leite EV | 1 | 0 | 1 | 0 | 0 | 2 |
| Misra PK | 1 | 0 | 0 | 0 | 1 | 2 |
| Ortiz JJ | 1 | 1 | 0 | 0 | 1 | 3 |
| Pengsaa K | 1 | 0 | 0 | 0 | 1 | 2 |
| Pine MC | 1 | 1 | 0 | 0 | 1 | 3 |
| Quiros-Buelna E | 1 | 0 | 0 | 0 | 1 | 2 |
| Romero-Cabello R | 1 | 0 | 0 | 0 | 1 | 2 |
| Sadjjadi SM | 1 | 0 | 0 | 0 | 1 | 2 |
| Yereli K | 1 | 0 | 0 | 0 | 1 | 2 |
| Canete R | 1 | 1 | 0 | 0 | 1 | 3 |
| Teles NSB | 1 | 0 | 0 | 0 | 1 | 2 |
| Almirall P | 1 | 1 | 0 | 0 | 1 | 3 |
| Canete R | 1 | 1 | 1 | 0 | 1 | 4 |
| 1 =yes; 0 = no | | | | | | |
