# Supplementary material for: Efficacy of 5-Nitroimidazoles for the Treatment of Giardiasis: A Systematic Review of Randomized Controlled Trials
Source: PLoS Negl Trop Dis. 2014 Mar 13;8(3):e2733. doi: 10.1371/journal.pntd.0002733 (PMC3953020; doi:10.1371/journal.pntd.0002733)
Supplement: Table S2 — Description of systematic reviews on drugs for treating giardiasis. (DOCX) [file pntd.0002733.s011.docx]

| **Table S2:** Description of systematic reviews on drugs for treating giardiasis | | | | |
| --- | --- | --- | --- | --- |
|  | **Zaat JOM *et al*** | **Solaymani-Mohammadi S *et al*** | **Granados CE *et al*** | **Pasupuleti V *et al*** |
| Year published | 1997 | 2010 | 2012 |  |
| Databases searched | Medline Index/Medicus, Embase, Cochrane Tropical Diseases Group Trial Register | PubMed, Scopus, Embase, Cochrane Controlled Trials Register, LILACS, ISI Web of Science | Cochrane Infectious Disease Group Specialized Register, the Cochrane Central Register of Controlled Trials (CENTRAL), MEDLINE, Embase, LILACS, the International Clinical Trials Registry Platform Search Portal | PubMed, the Cochrane Library, Scopus, ISI Web of Science |
| Inclusion criteria | Any trial of treatment of giardiasis in which a randomized comparison was attempted between drugs/regimens and with placebo or other drugs/regimens was included. Pseudo-randomized trials were also included. Patients should have laboratory proof for giardiasis at the start of treatment. | Included studies had to compare the effectiveness of albendazole with that of metronidazole in the treatment of giardiasis. Study had to allocate the study participants randomly to study groups. | RCTs comparing metronidazole administered for five to 10 days with any of the following drugs: metronidazole (single dose), tinidazole, albendazole, mebendazole, and nitazoxanide. | (i) RCTs evaluating the efficacy of 5-NI in comparison with a control (placebo, active, or another 5-NI); (ii) study population of patients with parasitologically-demonstrated giardiasis; (iii) study in any language. |
| Exclusion criteria | Open studies, case studies, double publications and studies with comparisons of treatment but without mentioning an attempt to randomize treatment allocation. Congress abstracts or unpublished reports are not included. | Duplicate publications, animal models of infections, studies of veterinary importance, studies *in vitro*, single-arm studies with no randomized control groups, studies lacking a comparison between the effectiveness of albendazole with metronidazole, review articles, studies with no clear randomization allocation procedure, studies using albendazole and metronidazole analogues as well as studies showing the synergistic effects between albendazole and/or metronidazole with other drugs. | Non-randomized trials, Data for primary outcome unavailable. | (i) No control group; (ii) efficacy data (parasitological cure rates) were not available or could not extracted for the study groups. |
| Method for exploring heterogeneity | Subgroup analysis of trials with symptomatic patients vs all patients; based on quality of the trials. | Subgroup analyses of trials which used least sensitive vs most sensitive methods to detect parasites; trials with clearly defined outcome measures. | Subgroup analyses: disease severity groups (ie hospitalized patients); participant age (children vs adults); pregnancy (yes  or no); co-infection (yes or no); HIV/AIDS (positive/negative);  cancer (yes or no); type of diagnostic methods;  country classification (low income, lower middle income, upper  middle income, and high income) | Subgroup analyses were pre-specified: (i) type of 5-NI used, (ii) excluding studies with two types of 5-NI comparisons, (iii) study setting (outpatient vs hospitalized), (iv) Jadad score (>3 vs <3), (v) type of main analysis (intention-to-treat vs per-protocol), (vi) sample size (<100 vs >100 patients), (vii) ordered by year of publication. |
| Method for evaluating quality of RCTs | Cochrane Collaboration guidelines | Jadad score | GRADE methodology | Jadad score |
| Primary outcome measures | Metronidazole vs other long treatments: cure rates OR 2.6, 95%CI 1.7-3.8; Tinidazole vs other short therapies: cure rates OR 5.5 95%CI 3.7-8.3 | Albendazole vs metronidazole: cure rates RR 0.97 95%CI 0.93-1.01 | Albendazole vs metronidazole: cure rates RR 0.99 95%CI 0.95-1.03 | 5NIs vs other drugs: cure rates RR 1.06, 95%CI 1.02-1.11, p=0.005 |
| Harmful outcome measures | Tinidazole vs other short therapies: diarrhea at follow-up OR 0.20 95%CI 0.08-0.50 | Albendazole vs metronidazole: adverse effects RR 0.36 95%CI 0.10-1.34 | Albendazole vs metronidazole: gastrointestinal side effects RR 0.29 95%CI 0.13-0.63; neurological side effects RR 0.34 95%CI 0.18-0.64 | 5NIs vs other drugs: abdominal pain RR 0.72, 95%CI 0.57-0.91; p=0.007; bitter or metallic taste RR 3.27, 95%CI 2.66-4.01; p<0.00001; headache RR 1.97, 95%CI 1.37-2.83; p=0.0003 |
